# Monitoring Training and Match Physical Load in Junior Soccer Players: Starters versus Substitutes

**DOI:** 10.3390/sports7030070

**Published:** 2019-03-19

**Authors:** Terje Dalen, Håvard Lorås

**Affiliations:** Faculty of Education and Arts, Department of Physical Education and Sport Science, Nord University, 7660 Levanger, Norway; havard.w.loras@nord.no

**Keywords:** soccer, training process, training load, soccer match, acceleration, sprint, high-speed running, Banister TRIMP

## Abstract

The aim of this study was to investigate differences in the physical (locomotor activities) and physiological (Banister’s training impulse) in-season training load between starters and substitutes in a well-trained junior soccer team. Physical performance variables from the Polar Team Pro system were collected and analyzed from a sample of junior soccer players (*N* = 18; age = 15.7 ± 0.5 years; stature, 177.9 ± 4.6 cm; body mass, 67.1 ± 5.5 kg). The study analyzed a total of 10 matches and 38 training sessions during the 2018 season with linear mixed models. The players from the starting line-ups demonstrated significantly higher average weekly physical load compared to the non-starters with respect to all variables: distance (total, running, high-speed running, and sprint) [F (1, 573) ≥ 66, *p* < 0.001, eta = 0.10], number of accelerations and sprints [F (1, 573) ≥ 66, *p* < 0.001, eta = 0.10], as well as Banister’s training impulse (TRIMP) [F (1, 569) = 10, *p* < 0.001, eta = 0.02]. Evidence from this study indicates that a large amount of weekly accumulated high-speed running and sprint distances is related to match playing time. Therefore, weekly fitness-related adaptations in running at high speeds seem to favor the starters in a soccer team.

## 1. Introduction

Understanding the physical demands of football (soccer) requires accurate and objective quantification of the players’ match activities [1,2,3,4]. It is well established that football is characterized by both low- (e.g., standing and walking) and high-intensity (e.g., running and sprinting) locomotor activities [5,6,7]. Along with football-specific behaviors such as tackles, turns, headers, and dribbles, these activities constitute the total physical load a player experiences during a match [8]. Recently, attempts have been made to quantify the total physical load through internal and external load variables, e.g., heart rate measurements, ratings of perceived exertion (RPE) and training impulse (internal), or by measuring locomotor variables through time–motion analysis systems (external) [1,9]. The external load is important for understanding the total work completed and the physical capacities of individual players, whereas the internal load is important for determining the physiological training load and subsequent adaptations [10]. As both external and internal loads are important for understanding overall training loads, a combination of both may be crucial in monitoring training [10]. The evolution of global positioning systems (GPS) now allows for valid and reliable estimates of the external load during both training and matches for soccer players. Furthermore, many of these systems also include heart rate measurement which allows to estimate the internal load accumulated by individual players.

Monitoring of training in soccer is applied for optimizing practice schedules and subsequent adaptations for increasing match performance. This monitoring allows the identification of the training status based upon both physiological and physical (locomotor distance and speed) variables. Moreover, the same monitoring could also be used to reduce the risk of non-functional overreaching which may lead to illness and injury [10]. Match play is typically associated with a higher amount of high-intensity running compared to training [11]. Moreover, one study conducted on professional soccer players demonstrated significant positive correlations between accumulated playing time in matches and aspects of physical performance, including sprint performance and muscle strength [11]. Discrepancies in both internal and external weekly accumulated load could lead to differences in important aspects of soccer-specific fitness between individuals based on different match playing times. Anderson et al. [12] reported that players who started a match generally covered longer running, high-speed running, and sprinting distances than non-starting players, and this difference was largely due to differences in accumulated playing time. Furthermore, a number of studies have shown that it seems difficult to re-create the high-intensity running associated with match play during training sessions, and this seems particularly true for high-speed running and sprinting [12,13]. Similarly, player accelerations in both training and matches need further research [14], as the number of accelerations is found to decrease throughout the match. Thus, it constitutes an important physical capacity that needs to be included in training schedules [15,16].

Impellizzeri and colleagues showed in a 2005 review that in Italian professional soccer players, one match in a week including five training sessions represents on average 25% of the total weekly internal training load (RPE-training load) [17]. The dominant role of the match in the weekly cycle with respect to both internal and external training loads seems to suggest that longer individual match playing time might favor those players (i.e., the starters) in improvement and maintenance of physical capacities relevant for soccer performance. As a consequence, starters and non-starters might demonstrate different physiological adaptations across time. For junior players, the adaptations across time would perhaps be more important given that matches are an important part of their soccer-specific practice. In addition, within these age groups, small differences in training load per week could amount to considerable differences across the complete training process from junior to senior level. However, most previous comparisons between starters and non-starters were made at the senior and elite levels. These soccer clubs have a second team where non-starters have considerable match opportunities, as well as resources to “take care” of the non-starters. Therefore, this study aimed to investigate differences between starters and non-starters in a club with only one U16 (15- and 16-year-old) boys team. Thus, the principal aim of this study was to investigate differences in physical (total distance, running distance, high-intensity running distance, numbers and distance of sprints, and numbers of acceleration) and physiological (Banister’s training impulse) in-season training load between starters and non-starters in a well-trained junior soccer team.

## 2. Materials and Methods

### 2.1. Subjects

The data for this study were collected from male junior soccer players from all outfield positions competing in the Norwegian junior league (*N* = 18; age = 15.7 ± 0.5 years; stature = 177.9 ± 4.6 cm; body mass = 67.1 ± 5.5 kg). Physical performance variables from a total of 10 matches and 38 training sessions during the 2018 season were collected and analyzed. The selected team is among the best-ranked junior soccer teams in the region. Players’ mean Yo-Yo intermittent recovery 1-test distance was 1580 ± 494.5 m, and average 20 m sprint time was 3.05 ± 0.1 s. Following an explanation of the procedures, all participants and parents gave verbal informed consent to participate in the study. The study was conducted according to the Declaration of Helsinki (2013).

### 2.2. Measurement

To monitor and evaluate the training load in junior soccer players, a Polar Team Pro tracking system based on GPS technology was applied to assess match and training loads of junior soccer players across 10 weeks. Players’ movements were measured by a sensor located on the chest, which continuously monitored the players’ heart rate, total distance, distance in different speed zones, and accelerations (https://www.polar.com/en/b2b_products/team_sports/team_pro). Speed, distance, and acceleration variables were registered at 10 Hz, whereas heart rate was sampled at 1 Hz. The following locomotor categories were selected for this investigation: running (from 11.0 to 14.9 km·h^−1^), High-Speed Running (HSR) (from 15.0 to 18.9 km·h^−1^), and sprinting (≥19.00 km·h^−1^). These speed zones were the default settings customized for soccer by the producer (https://www.polar.com/en/b2b_products/team_sports/team_pro). This HSR zone is classified as high-intensity running in other investigations, whereas the sprint zone is classified as very high-intensity running for elite male soccer players [4]. Acceleration was measured as the number of accelerations ≥ 2.0 m·s^−2^ [14]. After training and matches, the data were stored in a cloud-based server database for further off-line processing.

### 2.3. Procedures

Monitoring of the junior soccer players was done in-season during the months of August, September, and October. In total, this study tracked 10 weeks of training with one match (on Sundays) a week. Based on the players’ game-playing time in the match each week, players were divided into two separate groups: (1) Starters (Inclusion criteria: Played 60–80 min of 80 total min) and (2) Non-starters (Inclusion criteria: played 0–30 min of 80 total min). For the purposes of the current study, the training sessions included for analysis consisted of all the “on-pitch” training sessions each player was scheduled to undertake. The training load from strength training was not quantified in this study but it was similar between starters and non-starters (one training a week). The subjects had team practice 4 days a week (Tuesday–Friday). The data collection for the matches was carried out at both home and away grounds and at the club’s outdoor training pitch for the training sessions. The Banister’s training impulse (TRIMP) was calculated as described in Equation (1):

Training duration × ∆HR × 0.64*e*^1.92*x*^ (1)

where ∆HR equals (HR_exercise_ − HR_rest_)/(HR_max_ − HR_rest_), *e* is the Naperian logarithm having a value of 2.712, *x* equals ∆HR, and 1.92 is a constant for males [18]. The values for Banister’s TRIMP were expressed as arbitrary units (AU).

### 2.4. Statistical Analyses

Kolmogorov–Smirnov tests, histograms, and Q-Q plots were applied to confirm normality assumptions of the variables. The potential differences in physical load variables between matches/training and starters/non-starters were examined with linear mixed models, given that the current dataset violated the assumption of independence across measures (>1 datapoint for each player). In the analysis, match/training and starter/nonstarter were designated as fixed factors. In all pairwise comparisons, the alpha was Bonferroni-corrected, and the partial eta squared (η2p) was applied as a measure of effect size, where 0.01 < η2p < 0.06 constitutes a small effect, 0.06 < η2p < 0.14 constitutes a medium effect, and η2p < 0.14 constitutes a large effect [19]. Statistical analysis was conducted in PASW statistics 25.0 (IBM, New York, NY, USA), with *p* < 0.05 as a statistical significance criterion.

## 3. Results

### 3.1. Match versus Training

Across the entire sample, all physical load parameters were significantly (and substantially) higher in matches compared to training: total, running, high-speed, sprint distances [F (1, 573) ≥ 209, *p* < 0.001, eta ≥ 0.29], as well as number of sprints [F (1, 573) = 232, *p* < 0.001, eta = 0.27]. For these variables, one match included the same load as 1.6 times the training, 2.1 times the training, 2.4 times the training, and 3.4 times the training for total, running, high-speed, and sprint distances, respectively. In addition, the match load in number of sprints was similar to 2.3 times the training load. Significant, but smaller differences between matches and training were found in the number of accelerations [F (1, 573) = 4.64, *p* = 0.032, eta = 0.01] and Banister’s TRIMP [F (1, 573) = 25, *p* < 0.001, eta = 0.04]. For these variables, one match included the same load as 1.15 times the training and 1.3 times the training for acceleration and Banister’s TRIMP, respectively.

### 3.2. Starters versus Non-Starters: Total Physical Performance, Match, and Training

**Figure 1:**
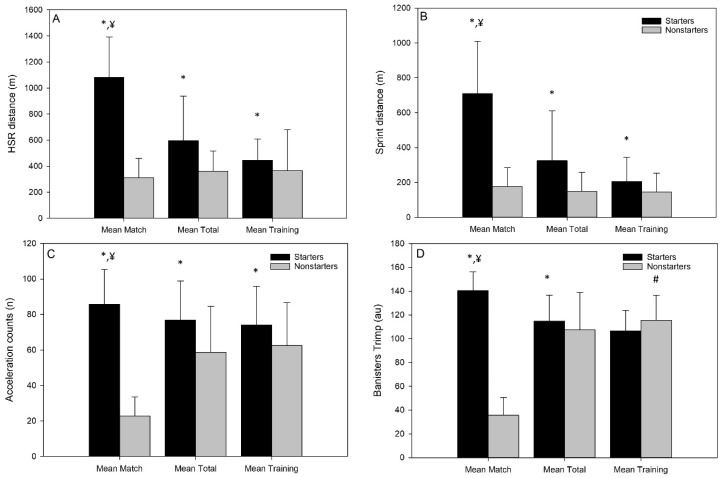
Weekly mean ± SD per session distance of high-speed running (**A**) and sprinting (**B**) for starters (black bars) and non-starters (grey bars) in match (mean match), in combined training session and match (total), and per training session in a week (training).Weekly mean ± SD per session number of accelerations (≥ 2.0 m·s^−2^) (**C**) and Banister’s training impulse (TRIMP) (**D**) for starters (black bars) and non-starters (grey bars) in match (mean match), in combined training session and match (total), and per training session in a week (training). * = higher in starters vs. nonstarters (*p* < 0.001), # = higher in nonstarters vs. starters (*p* < 0.001), ¥ = higher in match vs. training (*p* < 0.001).

As evident from Figure 1, the players from the starting line-ups demonstrated significantly higher average physical load compared to the non-starters in all variables: distances (total, running, high-speed running, and sprint) [F (1, 573) ≥ 66, *p* < 0.001, eta = 0.10], number of accelerations and sprints [F (1, 573) ≥ 66, *p* < 0.001, eta = 0.10], as well as Banister’s TRIMP [F (1, 569) = 10, *p* = 0.002, eta = 0.02].

As could be expected from the difference in accumulated match exposure, all physical load parameters from matches were significantly lower in the non-starters compared to the starters: total, running, high-speed running, and sprint distances [F (1, 108) ≥55, *p* < 0.001, eta ≥ 0.34], number of accelerations and sprints [F (1, 108) ≥ 39, *p* < 0.001, eta ≥ 0.26], as well as Banister’s TRIMP [F (1, 108) = 667, *p* < 0.001, eta = 0.86].

In the training data, the distance-related measures (total, running, high-speed running, and sprint) were all significantly higher in starters versus non-starters [F (1, 463) ≥ 15, *p* < 0.001, eta ≥ 0.03]. A similar pattern was found for number of sprints and accelerations [F (1, 463) ≥ 26, *p* < 0.001, eta ≥ 0.05]. However, training-related Banister TRIMP scores were statistically higher among non-starters compared to starters [F (1, 459) = 23, *p* < 0.001, eta = 0.05].

## 4. Discussion

The aim of the current study was to investigate differences in physical and physiological in-season training load between starters and non-starters in a junior soccer team. Our main findings were that the in-season physical training load was higher for starters than non-starters both in training and in matches. Training-related Banister’s TRIMP were higher among non-starters compared to starters. Further evidence from this study indicates that a large amount of weekly accumulated high-speed running and sprint distances was related to match playing time. Therefore, the weekly fitness-related adaptations in running at high speeds seem to favor the starters in a soccer team.

As evident from this study, small but significant differences in high-speed running, sprinting distance, and number of sprints between starters and non-starters were detected in training. These differences could be related to different factors and are in contrast to the findings by Anderson et al., who did not find differences between starters and non-starters in these variables [12]. Match play is typically associated with the highest amount of high-intensity running during a microcycle [12]. This will give starters a higher training load for high-intensity running weekly, which could influence soccer-specific fitness positively in order to create a higher degree of high-speed running in both matches and training. Thus, the high-speed running and sprinting demands seem difficult to recreate in training, e.g., with various small- or medium-sided games. Even large-sided games during training do not seem to fulfill the typical high-speed or sprint distance of official matches in elite soccer players [20]. Another factor for training differences in high-speed running and sprinting is that the starters may have higher soccer-related fitness and therefore, more substantial capacity for longer distances at high-speeds in training. Nevertheless, in order to bridge the gap between starters and non-starters, it seems important to pay attention to high-speed running variables in non-starters during training. In a previous investigation on high-speed and sprinting distances in different-sided games in training, Owen et al. found that high speed running and sprint distances were higher in large-sided games compared to medium- and small-sided games [20]. However, the distances run by elite male players at high speeds seemed to be lower in large-sided games than in official matches [21,22]. Although these findings were from elite male soccer players, the results from this study also suggest a substantially smaller amount of high-speed running and sprint distance in training compared to matches. In the current study, the sprint distances in matches for the starters were approximately the same as the sprint distances accumulated in weekly training. Therefore, additional training of high-speed and sprint running for non-starters might be important to consider in order to bridge the gap between starters and non-starters for these variables.

Our data show small differences between matches and training in the number of accelerations. For this variable, match performance was only 15% higher than the average training performance for starters. Recent studies have investigated the importance of accelerations in soccer match play. Akenhead et al. demonstrated that the number of accelerations is compromised following a peak period of activity: the peak acceleration period was on average 148% of the mean, and the number of accelerations in the following 5 min period was on average 10.4% lower than the mean [15]. Maintaining accelerations is very important for soccer performance, as up to 16% of the total player match load is caused by accelerations and decelerations [14], and a soccer match might contain as many as eight times more accelerations than sprints [4,23]. Nevertheless, unlike sprinting distance, the number of accelerations seems to be a considerable part of the training sessions. Indeed, recent findings indicate that small-sided games in training may have the same number of accelerations (>2 m·s^−2^) as peak periods of official matches [24]. Despite a difference in accelerations between starters and non-starters during training was confirmed, this difference had only a small effect. Similarly, the difference in accelerations between matches and training had a small effect in starters. Therefore, it seems possible for coaches to recreate peak periods of accelerations from matches during training in order to sufficiently stimulate adaptations.

Banister’s TRIMP was the only type of training load that was higher among non-starters compared to starters in this study. Although the difference was small, all other external training load variables were higher in starters compared to non-starters. Therefore, on the basis of the fact that a lower external training load induces a higher internal training load in non-starters, one may argue for a higher sport-specific fitness in the starters compared to the non-starters. Different small-sided games in training are useful in order to create high internal intensity (high percent of maximal heart rate) [25,26]. Thus, the fact that starters in the current study demonstrated a slightly higher TRIMP compared to non-starters in a weekly microcycle is related to match playing time. This in turn could cause different long-term physiological adaptations between the groups, even if the weekly physiological training load difference is small.

In conclusion, the results of the current study provide novel data on the challenge of conducting training sessions that provide high enough training load in high-speed running and sprinting to recreate the physical demands of matches. As a consequence of starters’ engagement in high-speed activity in match play, it seems to be of importance that training programs are designed specifically for non-starters in order to induce a training stimulus intense enough to create adaptations that maintain and improve the squad’s overall soccer-specific fitness. Additional training for non-starters for the high-speed variables seems very important, given that the sprint distance accumulated from one match represents 3.4 times the “on-pitch” soccer trainings. Less match playing time and no additional training in high-speed variables could thus potentially lead to reductions in the training status of non-starters with respect to this variable. Thus, coaches should be aware of and compensate for the discrepancy in the weekly training load for distances run at high-speeds between starters and non-starters.
